# Protein intake and transitions between frailty states and to death in very old adults: the Newcastle 85+ study

**DOI:** 10.1093/ageing/afz142

**Published:** 2019-11-11

**Authors:** Nuno Mendonça, Andrew Kingston, Antoneta Granic, Carol Jagger

**Affiliations:** 1 Institute for Ageing, Faculty of Medical Sciences, Newcastle University, NE2 4AX, UK; 2 EpiDoC Unit, CHRC, NOVA Medical School, Universidade Nova de Lisboa (NMS-UNL),1050 082, Portugal; 3 Institute of Health and Society, Faculty of Medical Sciences, Newcastle University, NE4 5PL, UK; 4 AGE Research Group, Institute of Neuroscience, Faculty of Medical Sciences, Newcastle University, NE2 4HH, UK; 5 NIHR Newcastle Biomedical Research Centre, Newcastle upon Tyne Hospitals NHS Foundation Trust, Newcastle University, NE4 5PL, UK

**Keywords:** malnutrition, aged 80 and over, multi-state model, PROMISS, fried, protein, frailty, older people

## Abstract

**Objectives:**

To examine the association of protein intake with frailty progression in very old adults.

**Design:**

The Newcastle 85+ study, a prospective longitudinal study of people aged 85 years old in Northeast England and followed over 5 years.

**Setting and Participants:**

668 community-dwelling older adults (59% women) at baseline, with complete dietary assessment and Fried frailty status (FFS).

**Measures:**

Dietary intake was estimated with 2 × 24-h multiple pass recalls at baseline. FFS was based on five criteria: shrinking, physical endurance/energy, low physical activity, weakness and slow walking speed and was available at baseline and 1.5, 3 and 5 years. The contribution of protein intake (g/kg adjusted body weight/day [g/kg aBW/d]) to transitions to and from FFS (robust, pre-frail and frail) and to death over 5 years was examined by multi-state models.

**Results:**

Increase in one unit of protein intake (g/kg aBW/d) decreased the likelihood of transitioning from pre-frail to frail after adjusting for age, sex, education and multimorbidity (hazard ratios [HR]: 0.44, 95% confidence interval [CI]: 0.25–0.77) but not for the other transitions. Reductions in incident frailty were equally present in individuals with protein intake ≥0.8 (HR: 0.60, 95% CI: 0.43–0.84) and ≥1 g/kg aBW/d (HR: 0.63, 95% CI: 0.44–0.90) from 85 to 90 years. This relationship was attenuated after adjustment for energy intake, but the direction of the association remained the same (e.g. g/kg aBW/d model: HR: 0.71, 95% CI: 0.36–1.41).

**Conclusion:**

High protein intake, partly mediated by energy intake, may delay incident frailty in very old adults. Frailty prevention strategies in this age group should consider adequate provision of protein and energy.

## Key points

- Most of the observed transitions were in the forward direction (i.e. robust to pre-frail and pre-frail to frail), but there were still some recoveries.
- Higher protein intake decreased the likelihood of incident frailty after adjusting for key socioeconomic and health factors, but not for other transitions (e.g. recovery from frailty).
- This relationship was attenuated after adjusting for energy intake but the direction of the association remained the same.

## Introduction

Frailty is a clinical syndrome defined as an increased vulnerability or failure to return to homeostatic equilibrium after a stressor event that increases the risk of dependency, hospitalisation and death [1]. Pre-frailty and frailty are estimated to be present in 42 and 11% of community-dwelling older adults, respectively, and both increase with age [2]. Moreover, frail older adults are at increased risk of disability, hospitalisation, care home admission and death [1]. Two popular frailty models include the cumulative deficits model [3] and the frailty phenotype [4], the latter using five criteria: muscle weakness, slow walking speed, low physical activity, exhaustion and unintentional weight loss [4]. Malnutrition is central to all the criteria proposed in the frailty phenotype [4]. Provision of adequate dietary protein could therefore be a viable strategy to modulate the progression of frailty in older adults [1] as it may slow down the progressive loss of muscle mass and physical function [5]. A recent systematic review concluded that older adults with higher protein intake were less likely to be frail but the studies included were mostly cross-sectional (prevalent frailty at baseline) as prospective studies with several time points were scarce and seldom included very old adults [6]. We have previously shown that low protein intake was associated with lower muscle strength and physical performance [7], worse disability trajectories [8] and incident disability [9] in very old adults. We therefore aimed to determine whether transitions between frailty states (robust, pre-frail and frail) and to death varied by protein intake in very old adults as they aged further. Our hypothesis was that higher protein intake was protective against frailty incidence between the age of 85 and 90 years but not impactful enough to promote recovery to either a pre-frail or robust state at this age.

## Methods

### Newcastle 85+ study

The Newcastle 85+ study is a longitudinal population-based study that approached all people turning 85 in 2006/2007 (born in 1921) in Newcastle and North Tyneside, UK. At baseline, the analytic sample comprised 668 very old adults living in the community, with complete protein intake assessment, height, weight and Fried frailty status (FFS). Full details of the Newcastle 85+ study have been published elsewhere [10].

### Protein intake

Dietary intake was assessed by a 24 h multiple pass recall on two non-consecutive occasions at baseline and nutritional intake estimated using the McCance and Widdowson’s sixth edition food composition tables [11]; full details can be found elsewhere [12, 13]. Body weight (BW) was measured to the nearest 0.1 kg using a digital scale and adjusted to be within the desired body mass index for older adults of 22–27 kg/m^2^ as previously described [14, 15]. Protein intake was expressed in three ways: as a continuous variable (g/kg aBW/d) and as a binary variable using cut points of 0.8 and 1.0 g/kg aBW/d based on previously published results from this cohort [7, 8, 15].

### Frailty

The FFS was derived for each time point based on approximations from the Cardiovascular Health Study methodology [4, 16] (Figure S1), by scoring (1) for every component that was present (shrinking, poor endurance/energy, low physical activity, weakness and slow walking speed) and (0) if absent (range 0–5). Further details of the individual components are given in the supplementary methods. Participants with a score of zero were defined as robust, with 1–2 as pre-frail and with 3 or more components as frail.

### Mortality and confounders

Information on date of death was obtained from National Health Service (NHS) Digital, UK [20]. The time to death was calculated as the time between age at baseline (2006–2007) and time of death (censored at 29 August 2012). Years of full-time education were categorised as 0–9 years, 10–11 years or 12 or more years in full-time education. Disease count was created by scoring eight chronic diseases (cardiac, respiratory and cerebrovascular disease, arthritis, hypertension, diabetes mellitus, cognitive impairment and cancer in the past 5 years) diagnosed by the General practitioner (GP) as either present (1) or absent (0) [17].

### Statistical analysis

Normality was assessed by Q–Q plots. Non-Gaussian distributed variables are presented as medians and interquartile ranges, and categorical data are presented as percentages (with corresponding frequency). To determine the contribution of protein intake (g/kg aBW/d) to transitions between FFS and to death over 5 years, we fitted a multi-state model with four states: robust, pre-frail, frail and death (absorbing state) (the illness-death model with the allowed transitions is shown in Figure S2). Due to the limited number of transitions between non-adjacent states (e.g. robust to frail) and the consequent non-convergence of the final models, we assumed that transitions from robust to frail or vice versa had to go through pre-frail.

We fitted three models for protein intake (g/kg aBW/d) (continuous) or protein intake ≥ or < to 0.8 (binary) or ≥ or < to 1 g/kg aBW/d (binary) with increasing complexity: Model l included protein intake, age, sex and years of full time education, Model 2 was further adjusted for number of chronic diseases from GP record reviews at baseline, 1.5, 3 and at 5 years of follow-up and Model 3 was also adjusted for energy intake.

Multi-state models describe the movement of an individual between a number of finite states in a continuous time stochastic process under the Markov assumption that the next state is only influenced by the current state [18, 19]. Multi-state models were fitted with the *msm* package in R v3.2.2 [20]. Point estimates and confidence intervals were used to assess statistical and clinical significance. The Broyden–Fletcher–Goldfarb–Shanno algorithm (quasi-Newton optimisation technique) was used to maximise the likelihood with results presented as hazard ratios (HR) and 95% confidence intervals (CI), alongside expected time spent in each state.

The Newcastle 85+ study was conducted according to the guidelines laid down by the 1964 Declaration of Helsinki, and all procedures involving human subjects were approved by the Newcastle and North Tyneside local research ethics committee (06/Q0905/2). Written informed consent was obtained from all participants, and when unable to do so, consent was obtained from a caregiver or a relative according to the UK Mental Capacity Act 2005.

## Results

### Missing and non-missing FFS

Compared to participants with a FFS, those with a missing FFS were in worse health and had lower protein intake. In addition, those without FFS were more likely to have missing data on protein intake and health variables (e.g. protein intake (g/kg aBW/d) was missing for 1% of the participants with a FFS and for 66% of those without) (Table S1).

### Baseline characteristics according to FFS

At baseline, women were more likely to be frail (e.g. 71.1% of those who were frail, and 47.3% of those who were robust were women) (Table 1) and this continued throughout the follow-up (Figure S1). Participants who were frailer at baseline had also more chronic diseases (e.g. 30.2% of frail and 8.5% of robust participants had four or more diseases). Those who were robust had, on average, higher energy intake and higher protein, carbohydrate and fat intake (but not percentage of energy from these macronutrients) than those who were pre-frail or frail (e.g. robust, pre-frail and frail participants had a protein intake of 66.7, 61.8 and 55.3 g/d, respectively) (Table 1).

**Table 1:** Baseline health and sociodemographic characteristics of participants in robust, pre-frail and frail FFS

	Robust (*n* = 129)	Pre-frail (*n* = 386)	Frail (*n* = 159)	All (*n* = 674)	Missing
Women	47.3 (61)	57.8 (223)	71.1 (113)	58.9 (397)	0 (0)
Education					0.3 (2)
0–9 years	60.5 (78)	62.8 (241)	71.7 (114)	64.4 (433)	
10–11 years	25.6 (33)	22.7 (87)	21.4 (34)	22.9 (154)	
12+ years	14.0 (18)	14.6 (56)	6.9 (11)	12.6 (85)	
Chronic diseases					0 (0)
0–1 diseases	35.7 (46)	32.1 (124)	13.2 (21)	28.3 (191)	
2–3 diseases	55.8 (72)	53.9 (208)	56.6 (90)	54.9 (370)	
4+ diseases	8.5 (11)	14.0 (54)	30.2 (48)	16.8 (113)	
Energy (MJ/d)	7.4 (6.2, 9.3)	6.8 (5.6, 8.0)	6.4 (5.2, 7.8)	6.8 (5.6, 8.3)	0.7 (5)
Total protein (g/d)	66.7 (53.1, 82.0)	61.8 (49.7, 75.2)	55.3 (44.8, 69.8)	61.2 (49.0, 76.0)	0.7 (5)
Energy protein (%)	15.5 (12.9, 17.4)	15.6 (13.5, 18.3)	14.9 (12.9, 17.4)	15.4 (13.2, 17.8)	0.7 (5)
Total protein (g/kg aBW/d)	1.0 (0.8, 1.2)	1.0 (0.8, 1.2)	0.9 (0.7, 1.1)	1.0 (0.8, 1.2)	0.9 (6)
<0.8 g/kg aBW/d	23.6 (30)	27.2 (104)	31.6 (50)	27.5 (184)	0.9 (6)
<1.0 g/kg aBW/d	47.2 (60)	54.0 (207)	62.7 (99)	54.8 (366)	0.9 (6)
Carbohydrate (g)	221 (172, 270)	190 (154, 231)	183 (153, 221)	192 (158, 236)	0.7 (5)
Energy carbohydrate (%)	48.0 (43.9, 54.0)	48.3 (43.3, 54.1)	49.5 (45.2, 53.2)	48.6 (43.9, 54.0)	0.7 (5)
Fat (g)	72.2 (57.7, 96.2)	63.7 (49.8, 79.7)	62.2 (47.2, 81.1)	65.0 (50.4, 83.9)	0.7 (5)
Energy fat (%)	36.4 (31.1, 41.6)	34.8 (30.5, 40.4)	37.3 (31.0, 41.1)	35.5 (30.8, 40.9)	0.7 (5)

Entries are percentages (%) and counts (*n*) for categorical variables and median (interquartile range) for non-normally distributed continuous variables. MJ, megajoules.

### Protein intake and transitions between frailty states and to death

**Figure 1:**
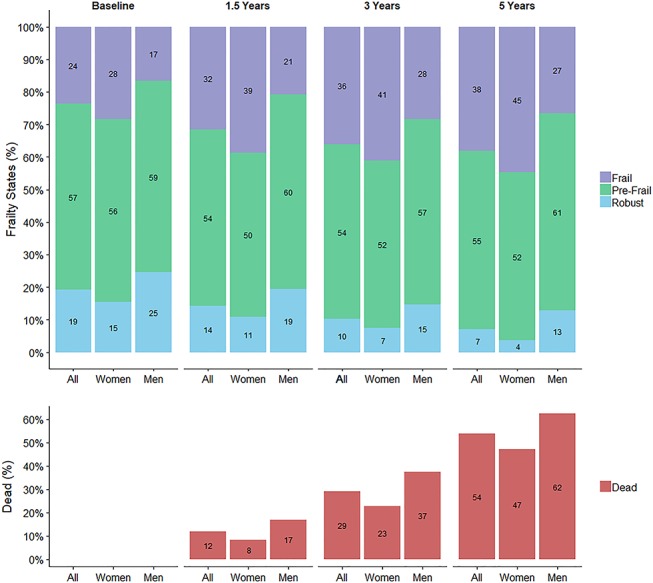
Fried frailty states and death (%), by follow-up and sex. Death is an absorbing state and is represented as a proportion of the total number of participants in each wave (cumulative).

There was a progressive decrease in robust FFS (19% of all participants at baseline, 7% by 5 years) and an increase in frail FFS (24% at baseline, 38% at 5 years) in men and women over the 5 years of follow-up (Figure 1). More than half (54%) of participants had died by 5 years. Table S2 shows the number of “transitions” between robust, pre-frail, frail and to death. On average, participants spent 1.43 years (95% CI: 1.15–1.77) robust, 3.01 years (95% CI: 2.63–3.44) pre-frail and 2.97 years (95% CI: 2.50–3.52) frail between age 85 and 90 years. An increase in one unit of protein intake (g/kg aBW/d) (continuous measure) decreased the likelihood of transitioning from pre-frail to frail in models adjusted for age, sex, education and number of chronic diseases (HR:0.44, 95% CI: 0.25–0.77) (Table 2). Significant reductions in incident frailty from pre-frailty were present in individuals with protein intake ≥0.8 (HR: 0.60, 95% CI: 0.43–0.84) and ≥1 g/kg aBW/d (HR: 0.63, 95% CI: 0.44–0.90) from 85 to 90 years (Table 2). These relationships were attenuated by further adjustment for total energy intake, but the direction of the associations remained the same (i.e. one unit increase g/kg aBW/d: HR: 0.71, 95% CI: 0.36–1.41; ≥0.8 g/kg aBW/d: HR: 0.67, 95% CI: 0.45–0.99; ≥1 g/kg aBW/d: HR: 0.78, 95% CI: 0.53–1.17) (Table 2). Other transition rates (robust to pre-frail, pre-frail to robust, pre-frail to dead and frail to pre-frail) did not vary by protein intake (Table 2), though there was a suggestion that participants with higher protein intake were less likely to die from a frailty state.

**Table 2:** Hazard ratios and 95% confidence intervals for the contribution of protein intake to transitions between FFS states and to death over 5 years

	Increase of 1 g/kg aBW/d		≥0.8 g/kg aBW/d		≥1 g/kg aBW/d	
	HR	95% CI	HR	95% CI	HR	95% CI
**Robust → pre-frail (*n* = 111)**						
Model 1^a^	0.99	0.51–1.93	0.75	0.48–1.19	1.29	0.89–1.88
Model 2^b^	0.96	0.49–1.87	0.75	0.47–1.20	1.30	0.89–1.91
Model 3^c^	0.87	0.37–2.04	0.69	0.41–1.16	1.32	0.87–2.00
**Pre-frail → frail (*n* = 173)**						
Model 1^a^	0.43	0.25–0.74	0.58	0.41–0.81	0.64	0.46–0.89
Model 2^b^	0.44	0.25–0.77	0.60	0.43–0.84	0.63	0.44–0.90
Model 3^c^	0.71	0.36–1.41	0.67	0.45–0.99	0.78	0.53–1.17
**Pre-frail → robust (*n* = 37)**						
Model 1^a^	1.25	0.48–3.21	0.87	0.37–2.01	1.76	0.84–3.68
Model 2^b^	1.16	0.45–2.97	0.81	0.34–1.91	1.74	0.82–3.68
Model 3^c^	0.85	0.24–3.07	0.65	0.25–1.69	1.65	0.70–3.87
**Pre-frail → dead (*n* = 140)**						
Model 1^a^	1.80	0.91–3.59	1.30	0.45–3.73	2.41	0.88–6.62
Model 2^b^	1.81	0.86–3.81	1.17	0.47–2.95	2.69	0.78–9.25
Model 3^c^	1.02	0.37–2.85	1.74	0.21–14.36	2.70	0.61–11.95
**Frail → pre-frail (*n* = 49)**						
Model 1^a^	1.02	0.38–2.75	0.70	0.36–1.36	1.20	0.66–2.18
Model 2^b^	0.99	0.37–2.68	0.64	0.32–1.28	1.20	0.66–2.19
Model 3^c^	0.79	0.21–2.92	0.48	0.21–1.12	1.20	0.61–2.34
**Frail → dead (*n* = 142)**						
Model 1^a^	0.60	0.34–1.06	0.79	0.57–1.10	0.63	0.43–0.93
Model 2^b^	0.62	0.34–1.11	0.84	0.60–1.17	0.61	0.41–0.91
Model 3^c^	0.85	0.41–1.77	0.90	0.61–1.34	0.65	0.41–1.02

Protein intake <0.8 or < 1.0 g/kg aBW/d was the reference category. *N* is the number of transitions.

^a^Model l included protein intake (g/kg aBW/d) or protein intake ≥ or < to 0.8 or ≥ or < to 1 g/kg aBW/d, age, sex and education.

^b^Model 2 was further adjusted for number of chronic diseases at baseline and follow-up.

^c^Model 3 was also further adjusted for energy intake.

### Sensitivity analysis

We tested for interactions between protein intake and energy but none were significant apart from the transition from pre-frail to dead (one unit increase g/kg aBW/d: HR: 0.48, 95% CI: 0.30–0.78). Conclusions remained after further adjustment for protein intake distribution throughout the day, smoking, alcohol intake and other macronutrients and with protein intake per actual body weight instead of aBW.

## Discussion

### Main findings

Participants with higher protein intakes at baseline were less likely to transition from pre-frail to frail between age 85 and 90 years, though this relationship was attenuated after adjusting for energy intake, suggesting that energy intake partly mediates the relationship between protein intake and frailty progression. To the best of our knowledge, this is the first study to investigate the contribution of protein intake to frailty incidence, recovery and transition to death in the very old.

### Protein intake and frailty incidence

Frailty is a complex construct, here operationalised by muscle weakness, slow walking speed, low physical activity, exhaustion and unintentional weight loss [4]. Nutrition is central to all these criteria [4], and higher protein is associated with a slower decline in grip strength [21, 22], muscle mass [23], walking speed [24] and weight-loss [25].

We found that participants with higher protein intake were less likely to have incident frailty (from pre-frailty) over 5 years in models adjusted for key confounders. These findings confirm a recent review concluding that higher protein intake was inversely associated with frailty in older adults (OR: 0.67, 95% CI: 0.56–0.82) [6], though only cross-sectional studies were included. Nevertheless, the few existing longitudinal observational studies, albeit mainly in younger old, are in agreement [6, 26]; older women (65–79 years) in the Women’s Health Initiative Observational Study (*n* = 24,000) with higher percentage of energy from protein (measured by food frequency questionnaire (FFQ)) were at reduced risk of frailty incidence (modified FFS) [27], and Spanish older adults (*n* = 1,800, 60+ years) with higher protein intakes (assessed by diet history) were less likely to develop incident frailty (FFS) over 3.5 years [28]. Conversely, others reported that older men (*n* = 5,900, 65+ years) from the Osteoporotic Fractures in Men study with higher percentage of energy from protein (assessed by FFQ) were as likely to be frail (modified FFS) after 4.6 years [29].

### Protein intake and other frailty transitions

Protein intake was not associated with other transition rates in our study (robust to pre-frail, pre-frail to robust, pre-frail to dead and frail to pre-frail) possibly because of insufficient transitions between each state. However, there was a trend in participants with higher protein intake to be less likely to die from a frailty state, confirming results from the Women’s Health Initiative where older women (65–85 years, *n* = 10,000) who were frail (modified FFS) and had higher biomarker-calibrated protein intakes (measured with FFQ and calibrated with recovery biomarkers of energy and protein in a subsample) were less likely to die over 12 years of follow-up (*P*-trend = 0.03) [30].

### Relationship partly mediated by energy intake

The observed associations between protein intake and frailty incidence were attenuated by further adjustment for energy intake though the direction of the associations remained, suggesting that energy intake partly mediates the relationship between protein intake and the transition between pre-frailty to frailty. This reinforces the key structural, functional and energy-producing role of protein. However, it remains difficult to disentangle the benefits of dietary protein and energy on frailty progression from previous studies since protein intake has been expressed as a fraction of energy on a number of occasions [27, 29]. Sufficient dietary energy is required for protein to optimally stimulate muscle protein synthesis and reduce the loss of muscle mass [31], and it may be that our observations are also a reflection of this.

### Strengths and weaknesses

FFS was assigned to participants at baseline, 1.5, 3 and 5 years, but unobserved incidence and recovery from frailty states may have occurred between these time points. However, sustained recovery (either from pre-frail or frail) was uncommon in the very old adults of the Newcastle 85+ study and therefore we can assume that most of these unobserved transitions were from either (i) robust to pre-frail or (ii) pre-frail to frail, or (iii) that recovery was transient and soon reversed. Protein intake was measured at baseline only and, therefore, intakes were assumed to be stable or have declined proportionally over 5 years. Furthermore, misreporting is a common limitation of self-reported dietary assessment methods. We estimated that 26% of the participants were possible misreporters (using an energy intake: basal metabolic rate cut-off of 1.05–2.00) but these were not excluded because of uncertainty surrounding this estimate and the small differences between excluding and including misreporters [11]*.* Healthy behaviours cluster together (non-smoking, higher physical activity, more balanced diet, etc.), and higher protein and energy intake could have served as a proxy for other behaviours negatively associated with frailty*.* Finally, although attempts were made to infer causality from our study (plausibility, temporality and consistency), this cannot be proved. However, our findings are supported by a small trial (87 frail older adults) where protein-calorie supplementation of 2 × 200 ml (400 kcal and 25 g of protein) liquid supplements per day over 12 weeks resulted in improved outcomes highly relevant to FFS (slower decrease in the short physical performance battery and usual gait speed and improved timed-up-and-go time) compared to the control group [32]. The large range of variables collected and the use of multi-state models to understand the contribution of protein to transitions between frailty states and to death over 5 years are major strengths of this study.

## Conclusion

Higher protein intake may delay the incidence of frailty in very old adults and this effect seems to be partly mediated by energy intake. This suggests that protein modulates frailty transitions not only because it provides energy but also for its functional and structural role. These findings need to be replicated in younger cohorts with long follow-ups where there is a greater possibility of recovery and where most participants start in a robust FFS and not already pre-frail/frail. Frailty prevention strategies in older adults should consider adequate provision of protein and energy.

## Supplementary Material

aa_19_0086_File004_afz142
