# Supplementary material for: Protein intake and transitions between frailty states and to death in very old adults: the Newcastle 85+ study
Source: Age Ageing. 2019 Nov 11;49(1):32–8. doi: 10.1093/ageing/afz142 (PMC6939283; doi:10.1093/ageing/afz142)
Supplement: aa_19_0086_File004_afz142 [file aa_19_0086_file004_afz142.docx]

***Protein intake and transitions between frailty states and to death in very old adults: The Newcastle 85+ Study***

*Supplementary Methods*

*Frailty*

BMI was calculated as BW (kg) divided by height^2^ (m) and used for the shrinking component. Poor endurance/energy was defined as present if the answer to “do you feel full of energy” was negative and the answer to “during the last 4 weeks how often did you rest in bed during the day” was “every day” or “every week”. Low physical activity was derived from a purposely designed physical activity questionnaire [[20](#_ENREF_20)]. Isometric grip strength (GS) was measured in kilograms (kg) using a Takei hand dynamometer and recorded twice for each hand. The four measurements were averaged and sex-specific GS and BMI cut-offs were used to define weakness [[7](#_ENREF_7)]. The time to complete the timed up-and-go test (participants were sat on a chair and had to get up and walk as quickly as possible to and around a 3m point and sit again) was used to define slow walking speed [[7](#_ENREF_7)] (more details in **Fig S1**).

| **Shrinking** | **Poor endurance/ energy** | **Low physical activity** | **Weakness** | **Slow walking speed** |
| --- | --- | --- | --- | --- |
| BMI < 18.5 kg/m^2^ | Geriatric depression scale item: answer to “do you feel full of energy” was “No” & the answer to “During the last 4 weeks how often did you rest in bed during the day” was “every day” or “every week” | Frequency of last moderately and very energetic physical activity was 1-3 times per month or less | Men  BMI≤ 24 & GS≤ 29  BMI 24.26 & GS≤ 30  BMI 26.1-28 & GS≤30  BMI>28 & GS≤ 32  Women  BMI≤ 23 & GS≤ 17  BMI 23.1-26 & GS ≤ 17.3  BMI 26.1-29 & GS≤ 18  BMI>29 & GS≤ 21 | TUG≥ 19 seconds |
| Each component that was present was scored as (1) and (0) if absent. Fried frailty status was derived by summing the five components: **Robust: 0**; **Pre-Frail: 1-2**; **Frail: 3-5** | | | | |

**Fig S1**. Fried frailty index operationalised for the Newcastle 85+ Study. Scores were imputed as Robust if total score was 0 with no item missing, as Pre-Frail if total score was 1 with one item missing, and as Frail if total score was 3 or 4 with 1 or 2 items missing. Questions for the poor endurance/ energy criteria were taken from the geriatric depression scale (15-items) and a social participation and social support questionnaire. BMI, body mass index; GS, grip strength; TUG, Timed-up-and-go.


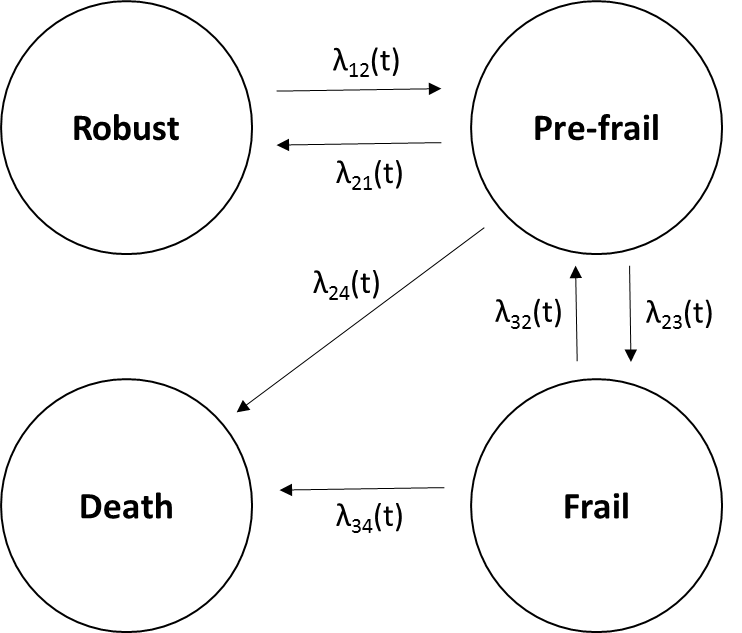


**Fig S2.** Illness-death multi-state model with the allowed transitions between Fried frailty status states.

**Table S1.** Baseline health and sociodemographic characteristics of participants with and without Fried frailty status.

|  | **FFS** (n=674) | Missing | **Non-FFS** (n=85) | Missing |
| --- | --- | --- | --- | --- |
| Women | 58.9 (397) | 0 (0) | 74.1 (63) | 0 (0) |
| Education |  | 0.3 (2) |  | 2.4 (2) |
| 0-9 years | 64.4 (433) |  | 62.7 (52) |  |
| 10-11 years | 22.9 (154) |  | 25.3 (21) |  |
| 12+ years | 12.6 (85) |  | 12.0 (10) |  |
| Chronic diseases |  | 0 (0) |  | 0 (0) |
| 0-1 diseases | 28.3 (191) |  | 18.8 (16) |  |
| 2 – 3 diseases | 54.9 (370) |  | 60.0 (51) |  |
| 4+ diseases | 16.8 (113) |  | 21.2 (18) |  |
| Energy (MJ/d) | 6.8 [5.6, 8.3] | 0.7 (5) | 6.3 [4.8, 8.0] | 37.6 (32) |
| Total protein (g/d) | 61.2 [49.0, 76.0] | 0.7 (5) | 56.3 [41.5, 70.4] | 37.6 (32) |
| Energy protein (%) | 15.4 [13.2, 17.8] | 0.7 (5) | 16.2 [12.6, 18.4] | 37.6 (32) |
| Total protein (g/kg aBW/d) | 1.0 [0.8, 1.2] | 0.9 (6) | 1.0 [0.7, 1.1] | 65.9 (56) |
| <0.8 g/kg aBW/d | 27.5 (184) | 0.9 (6) | 37.9 (11) | 65.9 (56) |
| <1.0 g/kg aBW/d | 54.8 (366) | 0.9 (6) | 58.6 (17) | 65.9 (56) |
| Carbohydrate (g) | 192 [158, 236] | 0.7 (5) | 176 [120, 228] | 37.6 (32) |
| Energy carbohydrate (%) | 48.6 [43.9, 54.0] | 0.7 (5) | 49.0 [43.4, 53.4] | 37.6 (32) |
| Fat (g) | 65.0 [50.4, 83.9] | 0.7 (5) | 58.9 [44.5, 76.3] | 37.6 (32) |
| Energy Fat (%) | 35.5 [30.8, 40.9] | 0.7 (5) | 36.6 [31.2, 41.0] | 37.6 (32) |

Entries are percentages (%) and counts (n) for categorical variables and medians (interquartile ranges) for non-normally distributed continuous variables. aBW, adjusted body weight; FFS. Fried frailty status; MJ, megajoules.

**Table S2.** Number of “transitions” between each FFS state and to death over 5 years.

|  | **To** | Robust | Pre-frail | Frail | Dead |
| --- | --- | --- | --- | --- | --- |
| **From** |  |  |  |  |  |
| Robust |  | 91 | 111 | 9 | 19 |
| Pre-frail |  | 37 | 453 | 173 | 140 |
| Frail |  | 0 | 49 | 210 | 142 |

FFS, Fried frailty status. For example, 91 participants remained robust over 5 years while 173 transitioned from pre-frail to frail.
